# Natural Killer Cell Therapy Combined with Probiotic Bacteria Supplementation Restores Bone Integrity in Cancer by Promoting IFN-γ Production

**DOI:** 10.3390/cells14171347

**Published:** 2025-08-29

**Authors:** Kawaljit Kaur, Patricia Reese, Jason Chiang, Anahid Jewett

**Affiliations:** 1 Division of Oral Biology and Medicine, The Jane and Jerry Weintraub Center for Reconstructive Biotechnology, Department of Dentistry, University of California Los Angeles (UCLA), Los Angeles, CA 90095, USA; patriciareese@ucla.edu (P.R.); ajewett@ucla.edu (A.J.); 2 Department of Radiology, Ronald Reagan UCLA Medical Center, Los Angeles, CA 90095, USA; cjchiang@mednet.ucla.edu; 3 The Jonsson Comprehensive Cancer Center, Los Angeles, CA 90095, USA

**Keywords:** supercharged NK cells, probiotic bacteria, pancreatic tumor, humanized-BLT mice, osteoclasts, IFN-γ

## Abstract

This study found a strong link between interferon-gamma (IFN-γ) secretion from immune cells and changes in bone quality in pancreatic tumor-bearing humanized-BLT (hu-BLT) mice. Tumor presence in hu-BLT mice led to bone resorption and reduced IFN-γ production compared to healthy mice. Interestingly, oral supplementation with probiotic bacteria AJ2, either alone or combined with supercharged NK (sNK) cells, inhibited tumor growth and increased IFN-γ levels in tissue compartments and tumor sites. Enhanced IFN-γ secretion was observed in cell cultures from the pancreas, spleen, PBMCs, splenocyte-derived NK cells, and bone marrow of mice treated with sNK cells and AJ2 compared to untreated tumor-bearing mice. Higher IFN-γ levels were associated with improved bone integrity in hu-BLT mice. TRAP staining showed increased osteoclastic activity and bone resorption in untreated tumor mice, in contrast to those treated with sNK and AJ2. This research highlights the role of immune cell-derived IFN-γ in preventing tumor-induced bone loss and improving bone quality, suggesting that probiotics, alone or with immunotherapies, have potential as treatments for osteolytic cancers.

## Simple Summary

The study explored the complex link between the immune system and bone structure, emphasizing the key role of IFN-γ in preserving skeletal integrity. Targeting the IFN-γ signaling pathway offers a promising way to strengthen bones and tackle cancer-induced bone disorders. It also highlighted the impact of supercharged NK (sNK) cells and probiotic bacteria in preventing tumor growth and spread in humanized-BLT (hu-BLT) mice. When probiotics are given alone or combined with sNK cell infusions, they boost IFN-γ secretion in hu-BLT mice, effectively stopping tumor-driven bone damage. This research points to the potential of probiotics, alone or with sNK cells, as innovative treatments for osteolytic cancers. It stresses the need to understand the mechanisms behind bone-tumor progression and calls for deeper insights into the bone marrow environment to combat cancer-related bone issues effectively.

## 1. Introduction

Over the last four decades, pancreatic cancer has remained one of the leading causes of cancer-related deaths worldwide, with little progress in improving the 5-year survival rate [1,2]. This aggressive form of cancer often spreads to organs like the liver, peritoneal cavity, lungs, brain, and bones, with reports of bone metastasis in pancreatic cancer dating back to 1963 [3]. Patients with pancreatic cancer often face challenges such as nutritional deficiencies, muscle wasting, and bone mass loss, leading to weakened bones and a higher risk of fractures due to cachexia [4]. Interferon-gamma (IFN-γ), a type II interferon cytokine, plays a vital role in various conditions like autoimmune diseases, infectious diseases, cancer immunology, and chronic inflammatory disorders [1,2,3]. Interestingly, IFN-γ levels tend to drop in peripheral blood and tumor microenvironment-derived NK and T cells in cancer patients [4,5,6]. Produced by innate and adaptive immune cells, this cytokine binds to constitutively expressed receptors, activating cell signaling and transcription through the *Jak1/STAT1* pathway [7,8,9,10,11,12,13]. IFN-γ production is finely controlled by other cytokines, impacting antiviral functions and several biological pathways [13]. In osteoimmunology, IFN-γ has a dose-dependent effect on bone formation and resorption [14,15]. Its influence on osteoclastogenesis and osteoclastic activity depends on dosage and experimental conditions, affecting immune responses against pathogens and tumors [16,17,18,19,20]. At low doses, IFN-γ supports osteoblast formation in both in vitro and in vivo studies, underscoring its importance in osteoblastogenesis [21].

Natural killer (NK) cells, crucial players in innate immunity, are instrumental in identifying and destroying tumor and virus-infected cells without prior sensitization [22,23,24]. NK cell-based immunotherapies have proven effective against various cancers [25,26,27,28,29,30]. However, utilizing these cells for cancer therapy faces obstacles such as their low abundance in peripheral blood (only 5–15% of peripheral blood mononuclear cells), impaired functionality in cancer patients, short lifespan (around 7 days), challenges in migrating to tumor sites—especially solid tumors—and limitations within the tumor microenvironment [4,31,32,33,34,35]. Recent studies reveal a complex relationship between NK cells and osteoclasts. NK cells influence osteoclast formation by expressing key factors like RANKL and M-CSF [36]. Furthermore, research shows that IFN-γ can inhibit RANKL, a critical regulator of osteoclasts, by promoting the ubiquitination and degradation of its signaling adaptor protein *TRAF-6* [7,8]. Interestingly, mice lacking IFN-γ or its receptor experience increased bone loss in collagen-induced arthritis (CIA) [37,38].

In this study, we utilized supercharged NK (sNK) cells, which are expanded NK cells induced by osteoclasts and probiotic bacteria. Previously, we demonstrated that combining osteoclasts with the probiotic bacteria AJ2 synergistically enhances NK cell activation and proliferation. Generating sNK cells is accomplished by co-culturing IL-2 and anti-CD16 mAb-treated primary NK cells with osteoclasts and sonicated AJ2 (sAJ2). sNK cells have demonstrated superior anti-cancer effects compared to primary NK cells [39]. When we characterized sNK cells, they expressed extended lifespans, retained function in the tumor microenvironment, and expressed increased cytotoxic granule expression and regulatory functions. With elevated activating receptors and reduced inhibitory receptor expression, their cytotoxic capabilities are significantly enhanced [40,41]. Our research highlights the exceptional anti-cancer potential of sNK cells, which outperform other NK cell treatments in cytotoxicity and cytokine secretion [40,42,43,44,45]. In vivo studies confirm their therapeutic promise, particularly in reducing pancreatic tumor cancer stem-like cells in hu-BLT mice [46]. The AJ2 formulation consists of seven gram-positive probiotic strains: *Streptococcus thermophiles*, *Bifidobacterium longum*, *Bifidobacterium breve*, *Bifidobacterium infantis*, *Lactobacillus acidophilus*, *Lactobacillus plantarum*, and *Lactobacillus paracasei*. These probiotics increase cytokine secretion, including IFN-γ, which promotes NK cell expansion [40,46,47,48,49]. Pancreatic cancer patients who consumed AJ2 orally (125 billion CFU/capsule: three capsules daily for four weeks) showed improved IFN-γ levels and cytotoxic activity in peripheral blood mononuclear cells (PBMCs) as well as in NK cells [50].

The gut microbiome plays a key role in bone health through various mechanisms. Probiotics improve nutrient absorption, immune function, and hormonal balance, aiding in the uptake of calcium, magnesium, vitamin D, and vitamin K, which are essential for bone strength [51,52,53]. Probiotics help regulate inflammation by balancing cytokines, reducing bone resorption [54]. Short-chain fatty acids from gut microbes support bone formation and increase bone density [55,56]. The effectiveness of probiotics depends on factors like how they are taken, the person’s bone health, and the specific strains used [57]. Studies suggest that probiotics can slightly reduce bone loss, particularly in postmenopausal women and the elderly, complementing calcium and vitamin D supplements [58,59]. Probiotics also influence the hormones and pathways involved in bone remodeling [60,61,62]. The link between the immune system and bone health, called osteoimmunology, is shaped by gut microbes, affecting immune and bone cell interactions [63,64]. Maintaining a healthy probiotic balance prevents inflammation that leads to bone loss and boosts immune function, benefiting skeletal health, especially for aging individuals or those with osteoporosis [65,66,67]. Overall, probiotics offer a well-rounded approach to improving bone health by enhancing nutrient absorption, reducing inflammation, and promoting bone formation.

Our research explores the impact of IFN-γ on bone health in cancer patients, utilizing sNK cells and probiotic bacteria AJ2 in pancreatic tumor-bearing hu-BLT mice. By using a humanized pancreatic tumor model with sNK cells and AJ2 supplementation, we aim to investigate the therapeutic effects on bone remodeling. The study highlights the role of IFN-γ in maintaining bone quality during cancer treatment, focusing on its influence on bone turnover and remodeling during tumorigenesis. We found that probiotic bacteria and sNK cells activate immune responses in pancreatic tumor-bearing hu-BLT mice, with oral administration of probiotics alone or combined with sNK cells significantly increasing IFN-γ levels in various tissues. Importantly, a strong link between IFN-γ production and bone formation was observed, showcasing its ability to prevent tumor-induced bone loss and promote regeneration. This research emphasizes the potential of sNK cells and AJ2 in enhancing IFN-γ levels to mitigate tumor-related bone degradation and support bone health in cancer patients.

## 2. Materials and Methods

### 2.1. Cell Lines and Reagents

Cell cultures of MiaPaCa-2 (MP2) tumor cells were cultured in DMEM media (Life Technologies, Carlsbad, CA, USA) with 10% Fetal Bovine Serum (FBS) (Gemini Bio-Products, West Sacramento, CA, USA). Cells isolated from hu-BLT mouse tissues were cultured in RPMI 1640 media (Life Technologies, CA, USA) supplemented with 10% FBS. Recombinant IL-2 (rh-IL-2) was purchased from Peprotech (Cranbury, NJ, USA) and used to activate immune cells. For osteoclast generation, peripheral blood mononuclear cells (PBMCs)-derived monocytes were cultured using alpha-MEM media (Life Technologies, CA, USA) with 10% FBS, along with M-CSF (Biolegend, San Diego, CA, USA) and RANKL (PeproTech, Cranbury, NJ, USA). A PKH26 staining kit was purchased from Sigma-Aldrich, St Louis, MO, USA.

### 2.2. Flow Analysis

Immune cells from hu-BLT mice were washed twice with ice-cold PBS containing 1% BSA. Flow cytometry analysis was conducted using a Beckman Coulter Epics XL cytometer (Brea, CA, USA), and the data were analyzed with FlowJo v10 software (Ashland, OR, USA).

### 2.3. Probiotic Bacteria AJ2 Formulation and Sonication

A patented mix of seven live probiotic strains—*Streptococcus thermophilus*, *Bifidobacterium longum*, *Bifidobacterium breve*, *Bifidobacterium infantis*, *Lactobacillus acidophilus*, *Lactobacillus plantarum*, and *Lactobacillus paracasei*—was formulated for oral feeding in hu-BLT mice, with a dosage totaling 5 billion CFU per dose. For sNK cell generation experiments, the strains were combined and suspended at 10 mg/mL in RPMI 1640 with 10% FBS. The process involved five sonication pulses, with each pulse including thorough vortexing and 15 s of ice incubation at amplitudes of 6 to 8. After sonication, the samples were incubated on ice for 30 s and checked under a microscope to ensure at least 80% bacterial cell wall lysis. About 20 cycles of sonication and ice incubation were carried out to achieve complete lysis. The sonicated probiotic samples were then aliquoted in RPMI 1640 media and stored frozen at −80 °C for later use.

### 2.4. Purifying Human NK Cells and Monocytes

PBMCs were used to isolate NK cells and monocytes through a negative selection method. The EasySep^®^ Human NK cell enrichment kit and monocyte isolation kit, both sourced from Stem Cell Technologies in Vancouver, BC, Canada, were utilized. NK cells and monocytes with purity levels over 94% were then used in the experimental procedures.

### 2.5. Generation of Osteoclasts and Supercharged NK Cells

Human peripheral blood-derived monocytes were cultured in alpha-MEM medium with M-CSF (25 ng/mL) for the first 3 days. From day 3 onward, the monocytes were maintained in alpha-MEM medium containing M-CSF (25 ng/mL) and RANKL (25 ng/mL) for 18 days, with the culture media refreshed every 3 days by replacing 70% with fresh medium containing M-CSF and RANKL. For supercharged NK (sNK) generation, freshly isolated NK cells were treated overnight with rh-IL-2 (1000 U/mL) and anti-CD16 mAb (3 μg/mL). These activated NK cells were co-cultured with osteoclasts in the presence of sonicated AJ2 (sAJ2) at a ratio of 1:2:4 (OCs:NK:sAJ2). RMPI 1640 supplemented with rh-IL-2 (1500 U/mL) was used to maintain the culture, with media refreshed every 3 days. The sNK cells were used for hu-BLT injection on day 15 of culture.

### 2.6. Tumor Implantation, sNK Cells Infusions, and AJ2 Feeding in hu-BLT Mice

All procedures involving hu-BLT mice were approved by the UCLA Animal Research Committee (ARC) and adhered to all federal, state, and local guidelines. Hu-BLT mice were created using NSG mice obtained from Jackson Laboratory [68,69] (Figure S1A). For pancreatic tumor hu-BLT mice experiments, mice were anesthetized using a combination of isoflurane and oxygen, and 1 × 10^6^ human MP2 pancreatic cancer stem-like cells in 10 μL HC Matrigel were implanted directly into the pancreas. One to two weeks after pancreatic tumor implantation, 1 × 10^6^ sNK cells were injected into the mice via the tail vein. For the AJ2 group, oral AJ2 feeding was started one week prior to pancreatic tumor implantation, and feeding was done every 48 h (5 billion CFU/dose) until the mice were euthanized. When the signs of morbidity appeared, the mice were euthanized, and pancreas/pancreatic tumor, bone marrow, spleen, and peripheral blood were collected.

### 2.7. Processing Tissues and Culturing Cells from hu-BLT Mice Tissue Samples

Single-cell suspensions of bone marrow were prepared by cutting the ends of the femurs and flushing them with RPMI 1640 medium. The collected bone marrow cells were filtered through a 40 µm cell strainer. Single-cell suspensions of spleen and liver were made by mincing the tissues and filtering the samples with a 40 µm cell strainer. The samples were centrifuged at 1500 rpm for 5 min at 4 °C, and the pellets were resuspended in ACK buffer for about 5 min to remove red blood cells. The final samples were resuspended in RPMI medium and centrifuged again at 1500 rpm for 5 min at 4 °C. The pancreas and/or pancreatic tumor samples were quickly cut into 1 mm^3^ pieces and placed into a digestion buffer containing 1 mg/mL collagenase IV, 10 U/mL DNAse I, and 1% bovine serum albumin (BSA) in DMEM media. Samples were incubated for 20 min in a 37 °C oven on a 150 rpm shaker. After digestion, the sample was filtered through a 40 µm cell strainer and centrifuged at 1500 rpm for 10 min at 4 °C. The pellet was then resuspended in DMEM media and cells were counted. Peripheral blood was collected in heparin, and single-cell isolation or PBMCs were obtained using Ficoll–Hypaque centrifugation, followed by resuspension in RPMI 1640 medium. Cell cultures from each tissue were maintained for 7 days by treating the samples with IL-2 (1000 U/mL) in RPMI 1640 medium. Serum was collected by incubating non-heparinized peripheral blood at room temperature for 20 min.

### 2.8. Isolating NK Cells from the Spleen of hu-BLT Mice

Splenocytes from hu-BLT mice were used to isolate NK cells with a human CD56+ selection kit (Stem Cells Technologies, Vancouver, BC, Canada). The NK cells were cultured for 7 days in RPMI 1640 medium supplemented with IL-2 (1000 U/mL).

### 2.9. Enzyme-Linked Immunosorbent Assay (ELISA)

IFN-γ secretion analysis of hu-BLT tissue cultures was carried out using a human ELISA kit (Biolegend, San Diego, CA, USA). The assays were conducted as recommended by the manufacturer and as described previously [70]. Absorbance values were measured at 450 nm using a microplate reader as specified in the Biolegend ELISA manual.

### 2.10. Bone Analysis

Micro-computed tomography (micro-CT) was used to examine bone architecture. Harvested samples were fixed in formalin, and high-resolution micro-CT images were captured at 10 µm resolution using the Skyscan 1275 system (Bruker microCT N.V., Kontich, Belgium). Data analysis was performed with the manufacturer’s software version 1.15, including Data Viewer, Recon, CTAn, and CTVol. Lumbar vertebrae (L3) from hu-BLT mice were dissected and fixed in 70% ethanol. Scanning was done with the Skyscan 1275 system equipped with a 5-µm focal spot micro-focus X-ray tube at 10 µm resolution (60 kVp, 166 mA, and a 1 mm Al filter). Specimens were aligned vertically within the scanner and stabilized with low-density foam in a 0.25-diameter tube. Phantom calibration was conducted to correlate micro-CT values with calcium hydroxyapatite (g/cm^3^). Images were reconstructed using NRecon software V2 for attenuation correction, ring artifact reduction, and beam hardening, then aligned in 3D with Data Viewer for accuracy. Segmentation involved manual comparisons of binarized and unsegmented images, applying a global threshold of 60. Irregular ROIs were manually drawn near the endocortical surface, with ROI lengths adjusted relative to vertebral height. For the third lumbar vertebrae, transverse micro-CT slices covered the entire vertebral body, and trabecular bone was analyzed within 0.5 mm of the growth plate. ROIs were sequentially drawn for each trans-axial micro-CT slice to ensure precision, and morphometric parameters were calculated from the binarized data.

### 2.11. Histology and Quantitative Histomorphometry

Static histomorphometry was performed on hu-BLT mice. Third lumbar vertebrae (L3) were dissected, fixed in 70% ethanol, dehydrated, and embedded undecalcified in methyl methacrylate. Frontal sections, 5 µm thick, were stained with 0.1% toluidine blue at pH 6.4. The static parameters of bone formation (OB) and resorption (OC) were measured within a defined area 0.25 mm from both growth plates and endochondral bone surfaces. Additional histochemical staining with tartrate-resistant acid phosphatase (TRAP) was conducted to identify osteoclasts.

### 2.12. Statistical Analysis

Linear Mixed Effects Models were used to analyze the micro-CT data to determine the differences between the comparisons (as differences in means between the groups). The linear model was used for the outcome on an interval scale (as opposed to a categorical scale), and a mixed-effects model was used to account for the correlation between the different outcomes within each mouse. For ex vivo data, for multiple groups, one-way ANOVA using Prism-10 software (Graphpad Prism, San Diego, CA, USA) was performed. The variable “n” denotes the number of mice used for each group. Duplicate or triplicate samples were used for each mouse. The following symbols represent the levels of statistical significance within each analysis: **** (*p*-value < 0.0001), *** (*p*-value 0.0001–0.001), ** (*p*-value 0.001–0.01), * (*p*-value 0.01–0.05). No stars shown indicate no significance.

## 3. Results

### 3.1. Successful Reconstitution of Human CD45+ Immune Cells in Tissues of hu-BLT Mice

The process of generating hu-BLT mice, shown in Figure S1A, included several detailed steps. To analyze the proportions of mouse and human immune cells in hu-BLT mice, flow cytometry was performed on single cells from various tissues, such as peripheral blood, spleen, BM cells, pancreas, and liver. The evaluation focused on murine and hu-CD45 expression levels. Human CD45 expression exceeded 71% in cells from the peripheral blood, spleen, and BM of hu-BLT mice (Figure S1B). In the pancreas, human CD45+ immune cells accounted for 15.6%, while 40.2% were observed in the liver (Figure S1B). Murine CD45 levels across all tissues ranged from 0.89% to 2.74%. These findings demonstrate the successful reconstitution of human immune cells in hu-BLT mice across various tissues, with varying levels of human CD45 expression.

### 3.2. Adoptively Transferred sNK Cells Migrate to Tissue Compartments of Pancreatic Tumor-Bearing hu-BLT Mice

**Figure 1:**
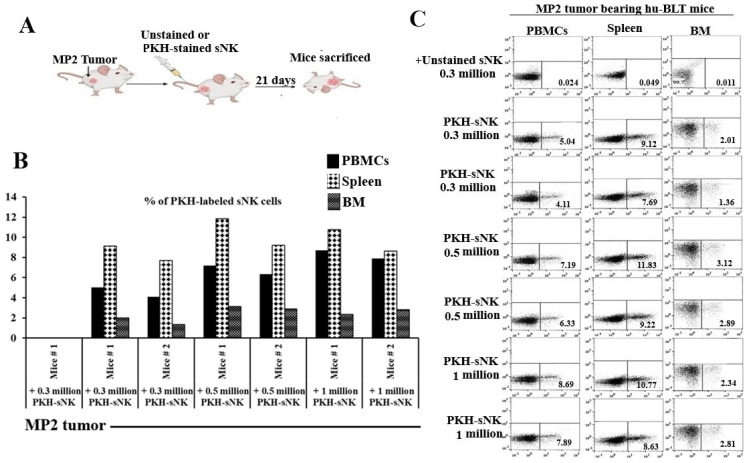
sNK cells persist and circulate in vivo within pancreatic tumor-bearing hu-BLT mice. Hu-BLT mice were implanted with 1 × 10^6^ MP2 in the pancreas, one week later, group 1 (n = 1) was adoptively transferred unstained 0.3 × 10^6^ sNK cells, group 2 (n = 2) was transferred 0.3 × 10^6^ PKH-sNK cells, group 3 (n = 2) was transferred PKH-0.5 × 10^6^ sNK cells, and group 4 (n = 2) was transferred 1 × 10^6^ PKH-sNK cells (**A**). sNK cells labeled with the fluorochrome PKH-26 were monitored via flow cytometry on day 21 in peripheral blood-derived PBMCs, spleen, and BM (**B**,**C**).

Our study concentrated on monitoring the biodistribution of adoptively transferred sNK cells across various tissue compartments in pancreatic tumor-bearing hu-BLT mice. The experiment included implanting MP2 pancreatic cancer stem cells (CSCs) in the pancreases of the mice, followed by administering PKH-labeled sNK cells, as shown in Figure 1A. We observed that the infused sNK cells traveled through the peripheral blood, spleen, and bone marrow, with their presence and distribution levels differing depending on the dosage administered (Figure 1B,C).

### 3.3. A Significant Rise in IFN-γ Secretion Was Observed in Mice Tissues When the Mice Were Infused with sNK Cells and Fed Probiotic Bacteria

**Figure 2:**
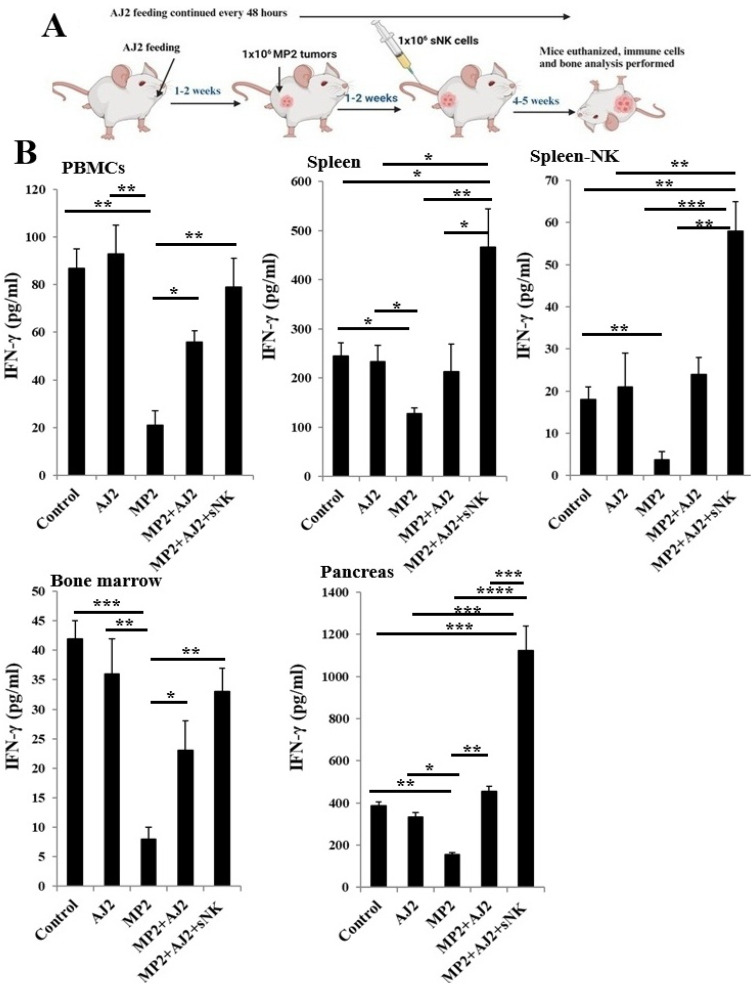
The combination of sNK cells and AJ2 boosted IFN-γ secretion by PBMCs, splenocytes, and bone marrow-derived immune cells, as well as tumor tissue-derived cells in hu-BLT mice. These mice were orthotopically injected with 1 × 10^6^ human MP2 tumors into the pancreas. One to two weeks post-tumor implantation, they received supercharged NK (sNK) cells via tail-vein injection and were orally administered 5 billion CFU AJ2 every 48 h, starting from one week before tumor implantation (**A**). At the experiment’s conclusion, the hu-BLT mice were sacrificed and their spleens, peripheral blood, bone marrow, and pancreas were harvested for single-cell suspension preparation. NK cells were isolated from splenocytes, and peripheral blood-derived PBMCs, splenocytes, spleen-derived NK cells, bone marrow-derived, and pancreatic cells were cultured with IL-2 (1000 U/mL) for 7 days. On day 7, supernatants were collected, and IFN-γ secretion was measured using a single ELISA (**B**). Results are shown as a bar graph, with data presented as Mean ± SD of three mice per treatment (n = 3). **** (*p*-value < 0.0001), *** (*p*-value 0.0001–0.001), ** (*p*-value 0.001–0.01), * (*p*-value 0.01–0.05).

Single cell isolations from different tissue compartments of hu-BLT mice were treated with IL-2 for seven days to evaluate IFN-γ secretion. IL-2 treatment was selected because of its well-known ability to boost IFN-γ gene transcription and secretion in immune cells, especially NK cells, T cells, and dendritic cells [71,72,73]. The experiments shown in Figure 2 highlight overall IFN-γ levels in immune cells from the tissues, without targeting a specific subset. Moreover, this data does not distinguish between infused sNK cells and endogenous immune cells. The main aim of these experiments was to determine whether probiotic bacteria supplementation, either alone or combined with sNK cell therapy, can enhance immune function, particularly restoring IFN-γ production, in tumor-bearing mice.

The study found no significant changes in IFN-γ secretion levels in tissues, including the pancreas, when mice without a tumor were fed probiotic bacteria AJ2 (Figure 2). However, in MP2 tumor-bearing mice, the presence of a pancreatic tumor caused a marked decrease in IFN-γ levels in PBMCs, spleen, spleen-purified NK cells, bone marrow, and the pancreas, the tumor site (Figure 2 and Figure S2). Interestingly, oral AJ2 administration restored IFN-γ levels in these tissues, especially at the tumor site (pancreas) (Figure 2 and Figure S2). Moreover, combining AJ2 feeding with sNK cell infusion significantly improved IFN-γ secretion in PBMCs and bone marrow. This approach also increased IFN-γ levels in serum, spleen, spleen-derived NK cells, and the tumor site (pancreas) (Figure 2 and Figure S2). These results emphasize the potential of AJ2 supplementation, alone or with sNK cell-based immunotherapy, to restore IFN-γ levels in immune cells across peripheral tissues and the tumor microenvironment.

### 3.4. Probiotic Bacteria-Fed hu-BLT Mice Showed Increased Bone Formation Compared to the Control Group

To examine the impact of probiotic bacteria on bone health, hu-BLT mice were fed 5 billion CFU probiotic bacteria AJ2 orally every 48 h for 4–5 weeks before being euthanized for bone analysis (Figure S3A). This resulted in enhanced bone formation compared to control mice. The analysis revealed increased bone volume (BV/TV) and trabecular number (Tb.n) in the AJ2-fed group. While comparisons of bone volume proportion, trabecular thickness, trabecular number, and trabecular spacing did not show statistically significant differences between the groups, higher Tb.Th and Tb.n values suggest elevated bone formation (Figure S3B). Additionally, smaller Tb.Sp values indicate a denser bone structure. Notably, 3D images demonstrated greater trabecular bone formation in AJ2-fed mice, supporting the overall trend of increased bone development in this group (Figure S3C). Although statistical significance was not observed, this data suggests improved bone formation in AJ2-fed hu-BLT mice, highlighting the potential of AJ2 probiotic supplementation for promoting bone health.

### 3.5. Using Probiotics as an Adjuvant Alongside sNK Cell Therapy Effectively Suppressed Pancreatic Tumor Growth in hu-BLT Mice

**Figure 3:**
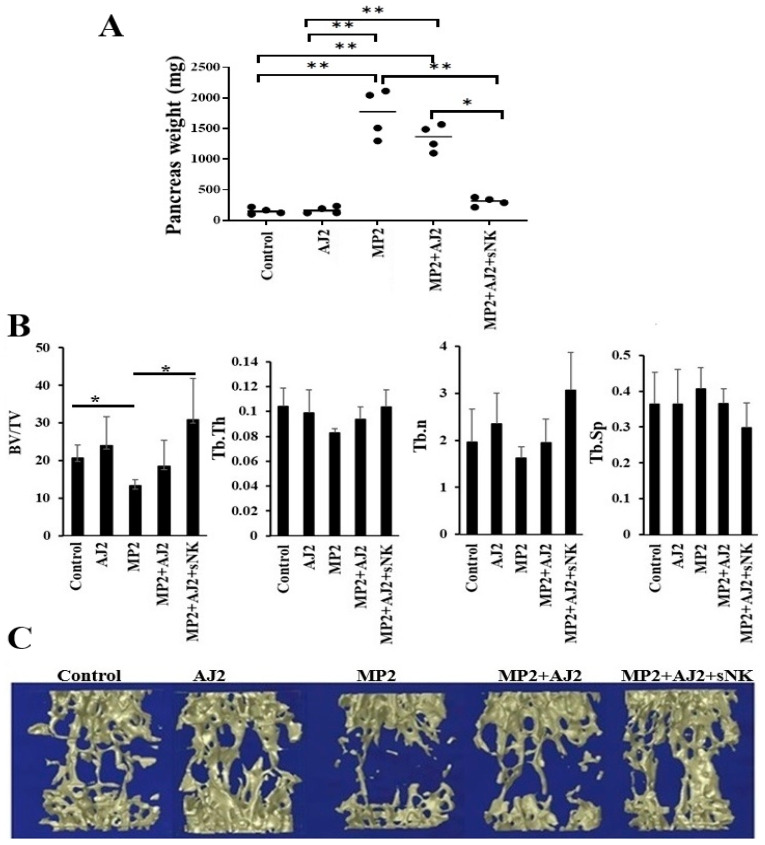
MP2 tumor-bearing mice treated with sNK cells and fed AJ2 showed significantly higher trabecular bone volume compared to the MP2 tumor and MP2 + AJ2 groups. Hu-BLT mice were orthotopically injected with MP2 tumors, treated with sNK cells, and fed AJ2 as described in Figure 2A. After the mice were sacrificed, the pancreas was removed and weighed in milligrams (mg) (n = 5) (**A**). Bone analysis was conducted as outlined in the Materials and Methods section. Bone volume/tissue volume (BV/TV), the percentage of bone volume within the total tissue volume, was calculated using the formula: (bone area/tissue area) × 100. Trabecular bone thickness (Tb.Th), a measure of trabecular bone microarchitecture in micrometers (µm), was calculated using the formula: (bone area/bone perimeter) × 2/1.2. Trabecular number (Tb.n), the number of trabeculae per millimeter, was determined using the formula: (BV/TV)/Tb.Th × 10. Trabecular separation (Tb.Sp), the average distance between trabeculae in micrometers (µm), was calculated using the formula: (1000/Tb.n) − Tb.Th. Higher values of Tb.Th or Tb.n indicate greater bone formation, while smaller Tb.Sp values suggest increased bone density. Results are shown as a bar graph, with data presented as Mean ± SD of five mice per treatment (n = 5) (**B**). ** (*p*-value 0.001–0.01), * (*p*-value 0.01–0.05). A 3D image of five representative lumbar vertebrae (L3) micro-CT scans is shown (**C**).

The study involved implanting MP2 pancreatic stem-like tumors into the pancreases of hu-BLT mice, feeding them probiotic bacteria AJ2, and adoptively transferring sNK cells, as shown in Figure 3A. Previous research has demonstrated the effectiveness of sNK cells in inhibiting tumor growth and restoring immune cell function in tumor-bearing hu-BLT mice [48,74,75]. After MP2 tumor implantation, tumor growth was observed in the pancreas. Mice fed only with AJ2 showed slower tumor growth, while those receiving the combined therapy had no palpable pancreatic mass. The mice were euthanized 4–5 weeks after sNK cell infusion, and their pancreas weight (in milligrams) was recorded. Control mice without tumors showed consistent pancreas weights, unaffected by AJ2 feeding (Figure 3A). In contrast, mice with MP2 tumors had a significant increase (6–10 fold) in pancreatic weight due to tumor growth. Tumor growth was slightly reduced with AJ2 feeding and significantly reduced with the combined sNK cell treatment (Figure 3A). This study highlights the promising potential of combining sNK cell therapy with probiotics to address pancreatic tumors.

### 3.6. Bone Formation Increased in Pancreatic Tumor-Bearing hu-BLT Mice Infused with sNK Cells and Given Probiotic Bacteria

MP2 pancreatic tumors caused a significant reduction in bone volume/tissue volume (BV/TV), indicating reduced bone volume fraction (Figure 3B). No stastistical significance was seen for trabecular thickness (Tb.Th), trabecular number (Tb.n), or increased trabecular spacing (Tb.Sp) compared to the control group (Figure 3B). But overall, reduced BV/TV, Tb.Th, and Tb.n and increased Tb.Sp indicates a lesser bone volume and higher bone resorption in pancreatic tumor mice. However, both healthy and tumor-bearing mice fed with AJ2 demonstrated improved BV/TV, Tb.Th, Tb.n, and Tb.Sp. Additionally, mice treated with sNK cells and fed with AJ2 showed further enhancements in BV/TV, Tb.Th, and Tb.n and reduced Tb.Sp compared to those on only AJ2 (Figure 3B, Figures S4A and S5A). These results were supported by 3D imaging, showcasing the interventions’ effects on the pancreatic tumor environment (Figure 3B, Figures S4B and S5B). IFN-γ secretion data mirrored the bone analysis, revealing lower bone formation levels and reduced IFN-γ secretion in MP2 tumor-bearing mice (Figure 2 and Figure 3B,C). Overall, AJ2 feeding or the combination of sNK cells and AJ2 restored IFN-γ secretion and effectively counteracted tumor-induced bone loss in this experimental model (Figure 2 and Figure 3B,C).

### 3.7. Increased Trabecular Bone Formation Was Observed in Pancreatic Tumor-Bearing Mice When They Were Fed Probiotic Bacteria, Either Alone or Combined with sNK Cell Infusions

**Figure 4:**
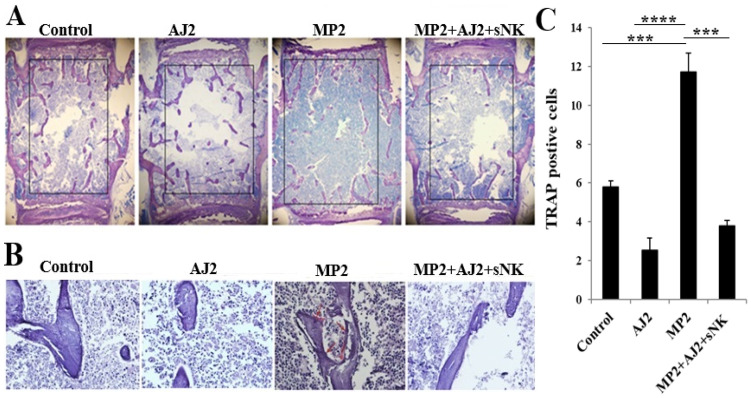
The AJ2-fed group showed increased bone formation compared to the control and tumor-bearing groups. Hu-BLT mice were orthotopically injected with MP2 tumors, treated with sNK cells, and fed AJ2 as outlined in Figure 2A. Histology and quantitative histomorphometry were conducted as detailed in the Materials and Methods section (n = 5) (**A**–**C**). Results are shown as a bar graph, with data presented as Mean ± SD of three mice per treatment (n = 3). **** (*p*-value < 0.0001), *** (*p*-value 0.0001–0.001).

To better understand the cellular process of bone remodeling, we conducted a histological analysis and static index assessments on the third lumbar vertebra. Consistent with the micro-CT findings and IFN-γ secretion data, immunohistochemistry revealed increased bone formation in AJ2-fed mice compared to control mice (Figure 4A). Similarly, enhanced bone formation was observed in MP2 tumor-bearing mice treated with a combination of sNK cell injections and AJ2 consumption, compared to untreated MP2 tumor-bearing mice (Figure 4A). In addition to micro-CT and IFN-γ analyses, we evaluated static bone resorption parameters, focusing on osteoclast activity using TRAP staining. Notably, TRAP-positive results were observed in samples from MP2 tumor-bearing subjects, whereas samples from the control group, AJ2-fed mice, and MP2-implanted mice treated with sNK cells and an AJ2 diet showed TRAP-negative results (Figure 4B,C). Overall, these findings indicate increased osteoclastic activity and bone resorption in the presence of tumors, compared to the control group or treated tumor-bearing mice (Figure 4B,C).

## 4. Discussion

In this study using humanized-BLT (hu-BLT) mice, we observed a significant reconstitution of human CD45+ immune cells in tissue compartments, as described in previous publications and this research [46,76,77,78] (Figure S1). The hu-BLT mouse model demonstrated that human stem-like tumors could promote tumor growth and metastasis [46,79,80]. Additionally, the adoptive transfer of supercharged NK (sNK) cells showed their ability to suppress tumor growth and enhance immune cell functionality, including IFN-γ secretion, across various cancer types in hu-BLT mice [46,81]. Importantly, the infusion of sNK cells exhibited a favorable safety profile, with no evident signs of toxicity in the hu-BLT mouse model [81]. This study further explored the pancreatic tumor hu-BLT mouse model to examine whether increased IFN-γ secretion contributed to bone restoration in tumor-bearing mice. The experimental process involved implanting an MP2 stem-like pancreatic tumor in hu-BLT mice, followed by administering PKH-labeled sNK cells to evaluate their distribution in the peripheral blood, bone marrow, and spleen of hu-BLT mice. Notably, the spleen displayed the highest accumulation of sNK cells, followed by PBMCs, with the lowest levels in the bone marrow (Figure 1). This uneven sNK cell distribution in the bone marrow may be due to the stromal bone marrow niche, which appeared to hinder NK cell activation, cytokine release, and their cytotoxic functions against cancer cells [82,83].

Cancer-associated bone loss is a complex condition influenced by factors like tumor metastasis, inflammation, and immune responses [84,85]. Breast, prostate, lung, kidney, and thyroid cancers often spread to the bones, disrupting the balance between bone formation and resorption [86,87]. Cancer metastasis leads to bone lesions, fractures, and pain. Cancer cells interact with the bone environment by releasing substances that increase bone resorption by osteoclasts, weakening the bone structure [87,88,89]. Inflammatory cytokines from bone cells can also promote tumor growth and bone degradation. Gut bacteria imbalances can further disrupt immune function, increase inflammation, and affect bone health, creating premetastatic niches in bones [90,91,92]. Probiotics like Lactobacillus and Bifidobacterium strains show immunomodulatory effects, reducing inflammation, restoring gut balance, and supporting immune function, making them valuable for addressing bone loss in cancer patients [54,93,94]. They help preserve gut health, prevent inflammation, and reduce bone resorption by strengthening the gut barrier, lowering endotoxemia, and preventing systemic inflammation from dysbiosis [54,95]. Animal studies highlight the benefits of L. acidophilus in preventing osteoporosis and inflammatory bone conditions through immune modulation and gut microbiota maintenance [94,96]. In oncology, probiotics show promise in reducing gut barrier damage and treatment-related inflammation, aiding in managing cancer-related bone loss [97,98,99].

Preclinical and early clinical research shows promise, but more well-controlled human trials are needed to identify the best probiotic strains, dosages, and integration methods for cancer care protocols aimed at preserving bone health [100]. The gut–bone axis influences bone remodeling through a complex network involving gut microbiota, immune cells like NK cells, and metabolic/endocrine pathways [51,101,102]. NK cells help regulate osteoclast and osteoblast activity via immune mediators shaped by gut microbiota signals, maintaining skeletal balance [103,104]. Strategies targeting gut microbiota and NK cell activity offer potential for restoring osteoclast/osteoblast balance and treating osteoporosis [105]. *Lactobacillus acidophilus* stands out for its immunomodulatory effects, which may prevent cancer-related bone loss by reducing inflammation, supporting gut integrity, and regulating osteoclast activity [106,107,108]. Probiotics, as adjunct therapies, show potential for cancer patients at risk of skeletal issues [98,109,110]. Combining probiotics with activated NK cells has shown promise in cancer models, enhancing IFN-γ secretion, inhibiting osteoclast activity, and supporting bone health [111,112]. Human trials suggest probiotics improve calcium absorption, vitamin D metabolism, and bone mineral density while lowering bone resorption markers [113,114,115]. These insights highlight promising strategies using probiotics to maintain bone health in cancer patients undergoing treatment.

In this study, we used the probiotic bacteria AJ2, which consists of seven gram-positive strains: *Streptococcus thermophilus*, *Bifidobacterium longum*, *Bifidobacterium breve*, *Bifidobacterium infantis*, *Lactobacillus acidophilus*, *Lactobacillus plantarum*, and *Lactobacillus paracasei*. When these probiotics were applied in vitro to NK cells, they significantly increased the secretion of both pro-inflammatory and anti-inflammatory cytokines and growth factors. Notably, IFN-γ and IL-1Ra levels were elevated, highlighting the enhanced activation of NK cells by probiotics, contributing to their anti-cancer properties [40,47]. In vivo studies with hu-BLT mice showed that combining sNK cell infusions with AJ2 feeding effectively inhibited pancreatic tumor growth and metastasis [46]. Tumor samples from hu-BLT mice treated with sNK cells and AJ2 feeding showed increased IFN-γ secretion and reduced IL-6 secretion [46]. Additionally, in pancreatic tumor-bearing hu-BLT mice, treatment with sNK cells, with or without AJ2 feeding, led to higher serum IFN-γ levels (Figure S2) [46]. These findings align with previous results, showing elevated IFN-γ levels in hu-BLT mouse tissues, including pancreatic cell cultures of MP2 tumor-bearing hu-BLT mice treated with sNK cells and AJ2 feeding, compared to untreated MP2 tumor-bearing and control mice [46] (Figure 2 and Figure S2).

Limited information exists on the in vivo effects of IFN-γ on bone tissue [18,116]. Vidal et al. emphasized the consistent anabolic effect of low doses of IFN-γ on bone, suggesting its potential as a therapy for bone loss [12,21]. Understanding IFN-γ’s impact on various cellular components in the bone marrow is key to evaluating its therapeutic potential for osteoporosis. IFN-γ’s regulation of bone cell differentiation and function has complex effects on skeletal health, with varying implications for pathological bone diseases [7,8]. It plays a role in regulating RANKL signaling and bone destruction by binding to osteoclasts, degrading RANKL signaling, and inhibiting bone resorption [38]. Studies indicate that IFN-γ can inhibit the osteoclast regulator RANKL by targeting *TRAF-6* for degradation, which can lead to greater bone loss in conditions like collagen-induced arthritis [7,8]. On the other hand, IFN-γ can promote osteoclast apoptosis by inducing superoxide production [117]. Its complex role also extends to conditions like osteopetrosis, where it influences immune system function rather than directly targeting bone cells [118,119,120]. In experimental bone metastasis models, Xu et al. showed that IFN-γ works in two ways: directly inhibiting tumor growth and reducing skeletal complications by modulating osteoclast function in host cells. This dual action highlights IFN-γ’s multifaceted role in bone health [121]. Research demonstrates that mice without functional IFN-γ signaling pathways are more susceptible to collagen-induced arthritis and related bone loss [122,123,124]. This vulnerability might stem from indirect effects on the host immune system rather than direct interactions with osteoclasts [125,126,127]. Studies also suggest an immunosuppressive state in cancer patients, indicating that compromised skeletal health and increased tumor-related bone loss in advanced cancer might be linked to a weakened immune system [122,123,124].

This study found a strong link between 3D images from micro-CT analysis, bone structure, and IFN-γ secretion levels. A reduction in IFN-γ secretion in the MP2-implanted hu-BLT group led to a significant decrease in bone volume, suggesting an impact on bone formation. This was further supported by decreased trabecular bone volume observed through 3D micro-CT analysis, histology, and histomorphometry. Bone parameter analysis showed a clear connection between bone integrity loss in the tumor-implanted mouse group and changes in bone volume fraction (BV/TV), trabecular thickness and numbers (Tb/Th and Tb/n), and trabecular separation (Tb/Sp). Interestingly, these changes were reversed when tumor-bearing mice received sNK cell infusion with AJ2 supplementation. These findings highlight the critical role of NK cells in maintaining bone integrity under pathological conditions. Additionally, the bacterial strains in AJ2 were specifically chosen to boost NK cell function and increase IFN-γ production, offering a promising strategy for addressing osteoporosis and bone loss. 

The study highlights the crucial role of IFN-γ in regulating osteoblastic and osteoclastic activities. Reduced IFN-γ levels hinder osteoblastic function while possibly enhancing osteoclastic activity, resulting in decreased bone volume. IFN-γ also exhibits direct anti-tumor effects, combating tumor-induced bone loss and demonstrating its dual role in supporting bone health and suppressing tumors. The research found a positive link between immune cell secretion of IFN-γ and improved bone formation, backed by micro-CT findings. AJ2 feeding in control and tumor-bearing mice boosted bone formation compared to their counterparts, emphasizing the potential of IFN-γ in promoting bone health. Tumor-bearing mice showed increased osteoclast activity and bone resorption, as revealed by TRAP staining, highlighting the detrimental impact of tumors on bones. Elevated IFN-γ levels appeared to counteract this tumor-induced bone loss, showcasing its therapeutic promise in addressing cancer-related bone problems. IFN-γ is key to maintaining bone marrow balance and immune-related bone health, as demonstrated in studies using neutralization or knockout models [128,129]. The absence of IFN-γ can alter immune responses, blood cell production, and bone cell activity, potentially helping to reduce immune-driven bone marrow failure [130,131,132]. However, IFN-γ-induced effects on bone remodeling and overall health can vary depending on the inflammatory environment [128]. Therapeutic approaches targeting IFN-γ must thoughtfully address its complex and sometimes contradictory roles.

Our findings underscore the complex relationship between the immune and skeletal systems, emphasizing IFN-γ’s essential role in maintaining skeletal health and its potential as a target for enhancing bone integrity and treating bone-related conditions. In previous studies, AJ2 alone, sNK, and their combination were tested, showing an additive effect on tumor inhibition and immune cell activation, including IFN-γ secretion from hu-BLT mice tissue-derived cells [46,48]. While recognizing that including AJ2 alone and sNK alone in this research would strengthen the conclusions, this study represents our initial bone analysis in tumor-bearing hu-BLT mice to assess the efficacy of AJ2 and/or sNK cell therapy in addressing tumor-induced bone defects. The combined approach was chosen as it proved most successful in reducing tumor growth and restoring immune function compared to AJ2 alone or sNK cells alone. Building on the favorable outcomes of this investigation, further exploration will involve examining AJ2 alone, sNK alone, and their combination at varying dose levels to establish the minimal effective, maximum tolerable, and optimal therapeutic values for managing tumor-induced bone loss.

## 5. Conclusions

The report highlights the importance of understanding the factors driving tumor progression in bone and emphasizes the need for more research into the bone marrow microenvironment to address cancer-associated bone problems. The study showcases the role of sNK cells and probiotic bacteria in reducing tumor growth and metastasis. Additionally, oral administration of probiotic bacteria, either alone or with sNK cell infusions, enhances IFN-γ secretion in hu-BLT mice, helping to prevent tumor-related bone damage. The findings suggest the therapeutic potential of probiotics, either on their own or in combination with immunotherapies, encouraging further investigation into treatments for osteolytic cancers. A lack or neutralization of IFN-γ may increase osteoclast activity, leading to bone loss due to missing inhibitory signals, while boosting IFN-γ production could offer therapeutic benefits for disorders related to bone loss.

## Data Availability

The original contributions presented in this study are included in the article/Supplementary Material. Further inquiries can be directed to the corresponding author.

## Supplementary Materials

The following supporting information can be downloaded at: https://www.mdpi.com/article/10.3390/cells14171347/s1, Figure S1: Reconstitution of human CD45+ immune cells in humanized-BLT (hu-BLT) mice tissues; Figure S2: Combination of sNK cells and AJ2 increased IFN-γ secretion by immune cells in hu-BLT mice; Figure S3: Mice fed with AJ2 presented increased trabecular bone formation when compared to the control group; Figure S4: MP2 tumor-bearing mice injected with NK cells and fed with AJ2, presented statistically significantly higher trabecular bone volume when compared to the control and MP2 + AJ2 group, respectively; Figure S5: MP2 tumor-bearing mice injected with NK cells and fed with AJ2, presented statistically significantly higher trabecular bone volume when compared to the MP2 tumor and MP2 + AJ2 group, respectively.

## Abbreviations

NK cells	Natural killer cells
sNK cells	Supercharged NK cells
CSCs	Cancer-stem-like-cells
Hu-BLT	Humanized-bone marrow/liver/thymus
IFN-γ	Interferon-gamma
IL-2	Interleukin 2
OCs	Osteoclasts
PBMCs	Peripheral blood-derived mononuclear cells
MP2	MiaPaCa-2
ELISAs	Enzyme-Linked Immunosorbent Assays
