# Supplementary material for: Natural Killer Cell Therapy Combined with Probiotic Bacteria Supplementation Restores Bone Integrity in Cancer by Promoting IFN-γ Production
Source: Cells. 2025 Aug 29;14(17):1347. doi: 10.3390/cells14171347 (PMC12427929; doi:10.3390/cells14171347)
Supplement: Supplementary file 1 [file cells-14-01347-s001.zip › cells-3800321-supplementary.pdf]

Supplementary data

Figure S1. Reconstitution of human CD45+ immune cells in humanized-BLT (hu-BLT) mice tissues. Hu-BLT mice were generated as described in Materials and Methods and shown in Figure (A). Reconstitution of human immune cells was determined in hu-BLT mice spleen, PBMCs, BM, pancreas, and liver using anti-human and anti-mouse CD45 and analyzed by flow cytometry (B).

Figure S2: Combination of sNK cells and AJ2 increased IFN-γ secretion by immune cells in hu-BLT mice. Hu-BLT mice were orthotopically injected with MP2 tumors, injected with sNK cells, and fed with AJ2. At the end of the experiment, hu-BLT mice were sacrificed, and the spleens, peripheral blood, pancreas, and bone marrow were harvested, and single-cell suspensions were prepared. Serum was harvested from peripheral blood, and the secretion of IFN-γ was determined using a single ELISA. NK cells were isolated from splenocytes. Peripheral blood-derived PBMCs, splenocytes, spleen-derived NK cells, bone marrow-derived cells, and pancreatic cells were cultured in the presence of IL-2 (1000 U/ml) for 7 days. On day 7, the supernatants were harvested, and the secretion of IFN-γ was determined using a single ELISA. The average of three representative experiments is shown as a bar graph, and data are presented as Mean±SD. ****(p value <0.0001), ***(*p* value 0.0001-0.001), **(*p* value 0.001-0.01), *(*p* value 0.01-0.05).

Figure S3: Mice fed with AJ2 presented increased trabecular bone formation when compared to the control group. Hu-BLT mice were orally fed with 5 billion AJ2 every 48 hours (A). Bone analysis is performed as described in the Materials and Methods section (B-C).

Figure S4: MP2 tumor-bearing mice injected with NK cells and fed with AJ2, presented statistically significantly higher trabecular bone volume when compared to the control and MP2+AJ2 group, respectively. Hu-BLT mice were orthotopically injected with 1 x 10^6 human MP2 tumors in the pancreas. One week after the tumor implantation, mice received supercharged NK (sNK) cells via tail-vein injection. Mice were orally fed with 5 billion AJ2 every 48 hours. Bone analysis is performed as described in the Materials and Methods section (A-B).

Figure S5: MP2 tumor-bearing mice injected with NK cells and fed with AJ2, presented statistically significantly higher trabecular bone volume when compared to the MP2 tumor and MP2+AJ2 group, respectively. Hu-BLT mice were orthotopically injected with 1 x 10^6 human MP2 tumors in the pancreas. One week after the tumor implantation, mice received supercharged NK (sNK) cells via tail-vein injection. Mice were orally fed with 5 billion AJ2 every 48 hours. Bone analysis is performed as described in the Materials and Methods section (A-B).

Figure S1

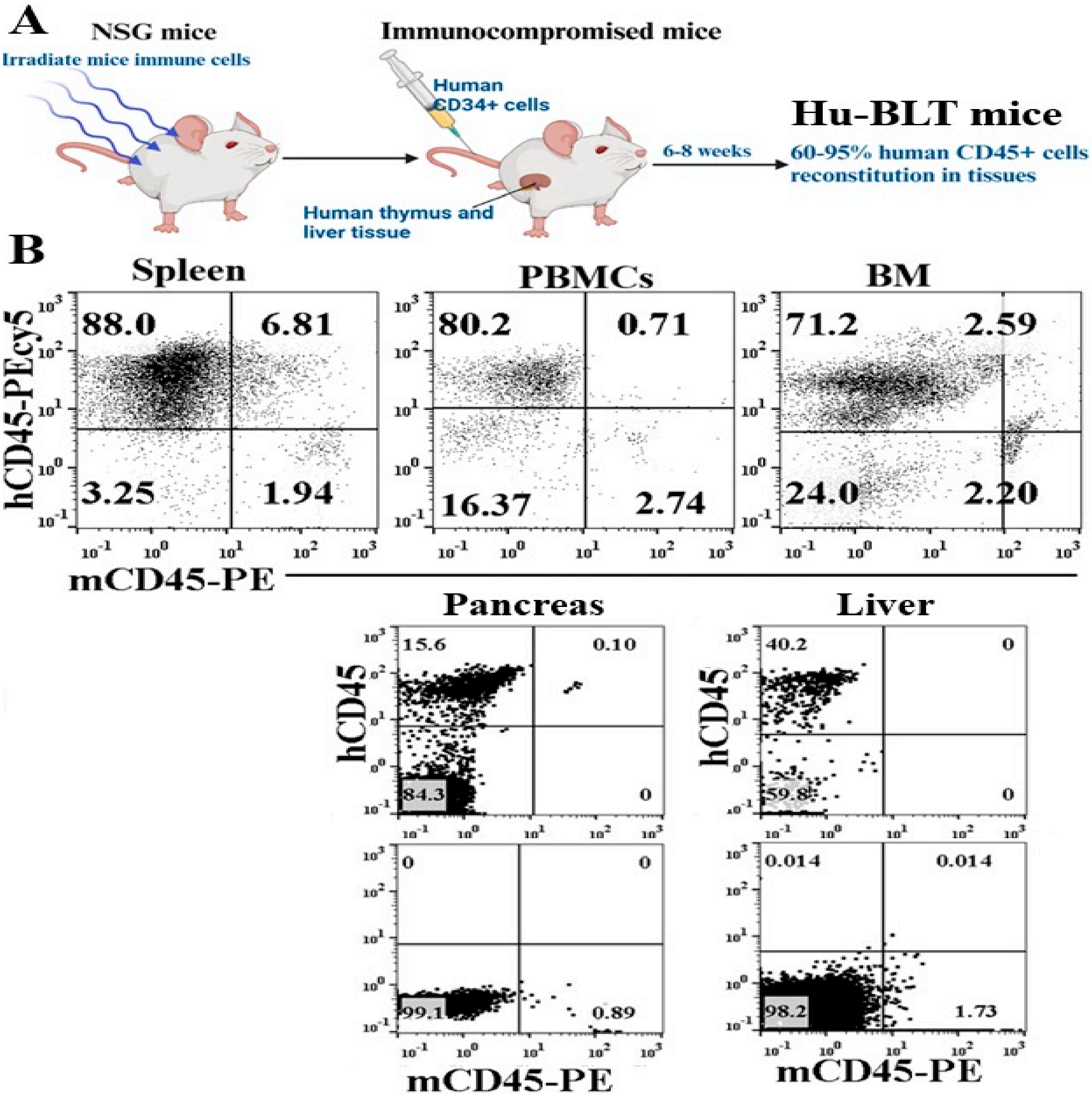

A
NSG mice
Irradiate mice immune cells
Immunocompromised mice
Human
CD34+ cells
Human thymus and
liver tissue
6-8 weeks
Hu-BLT mice
60-95% human CD45+ cells
reconstitution in tissues
B
Spleen
PBMCs
BM
hCD45-PEcy5
88.0
6.81
3.25
1.94
80.2
0.71
16.37
2.74
71.2
2.59
24.0
2.20
mCD45-PE
Pancreas
Liver
hCD45
15.6
0.10
84.3
0
0
0
99.1
0.89
40.2
0
59.8
0
0.014
0.014
98.2
1.73
mCD45-PE
mCD45-PE

Figure S2

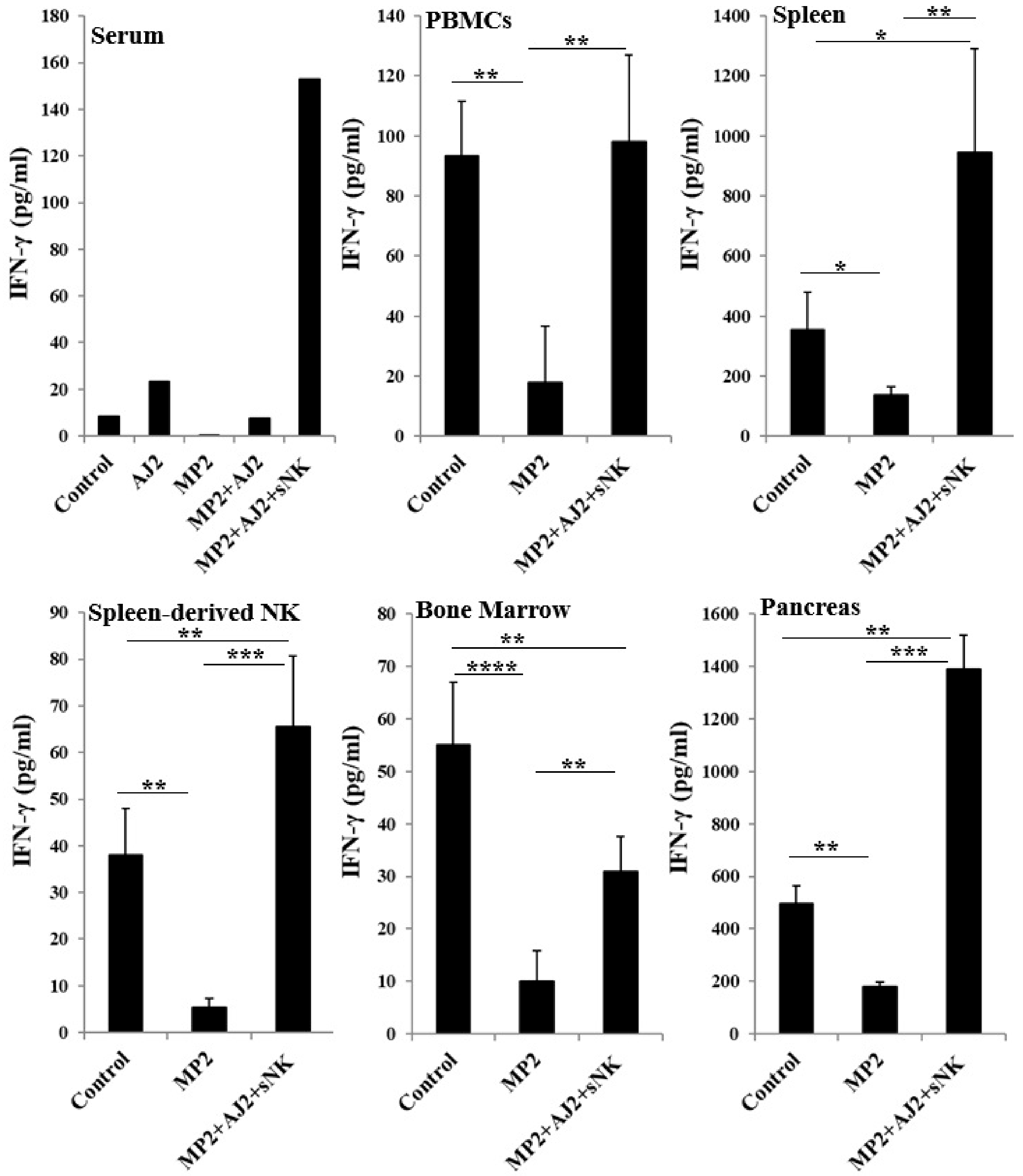

Serum
PBMCs
Spleen
Spleen-derived NK
Bone Marrow
Pancreas
IFN-γ (pg/ml)
Control
AJ2
MP2
MP2+AJ2
MP2+AJ2+sNK

Figure S3

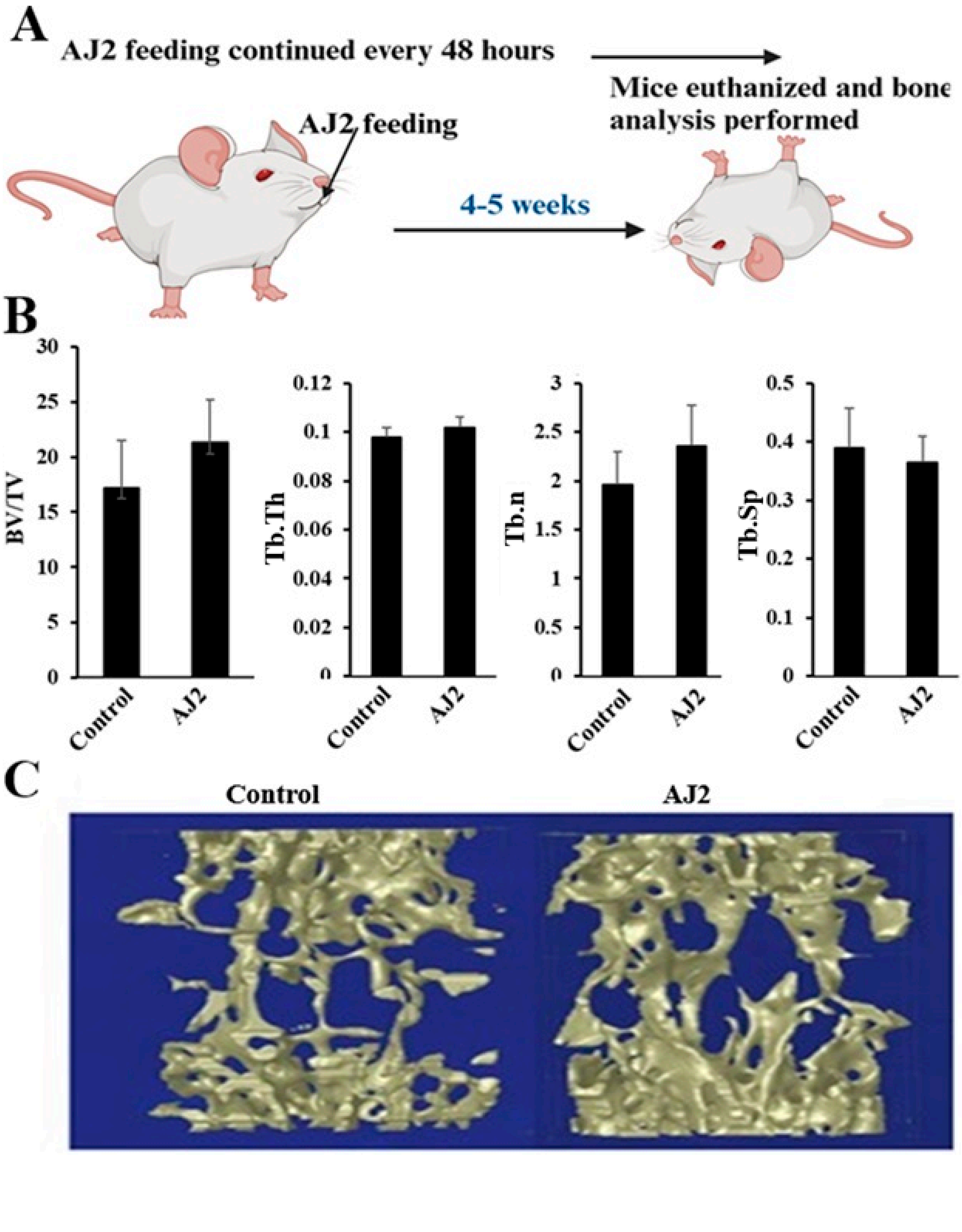
A
AJ2 feeding continued every 48 hours
Mice euthanized and bone analysis performed
AJ2 feeding
4-5 weeks
B
BV/TV
Tb.Th
Tb.n
Tb.Sp
Control
AJ2
C
Control
AJ2

Figure S4

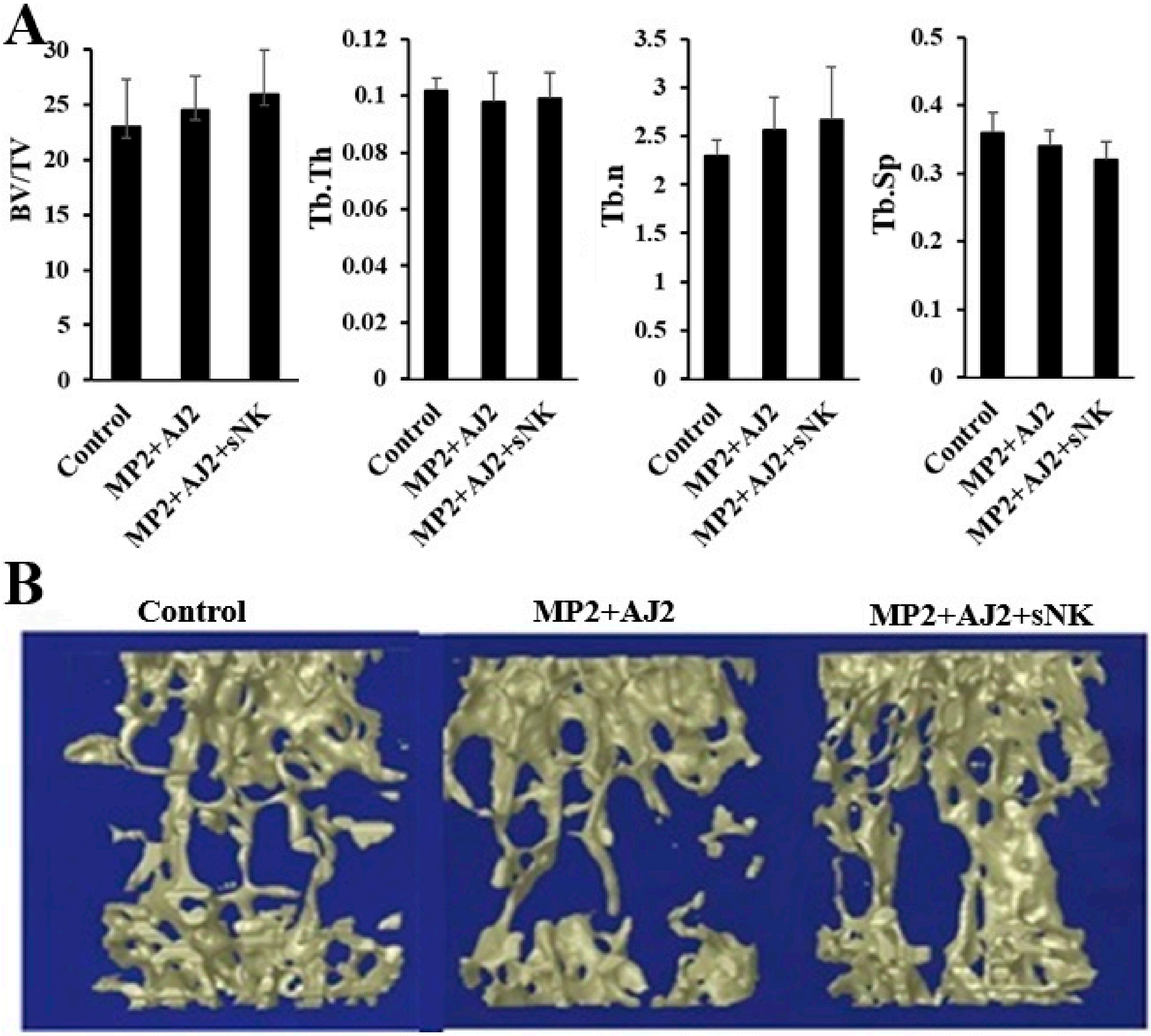
A
30
25
20
15
10
5
0
BV/TV
Control
MP2+AJ2
MP2+AJ2+sNK
0.12
0.1
0.08
0.06
0.04
0.02
0
Tb.Th
Control
MP2+AJ2
MP2+AJ2+sNK
3.5
3
2.5
2
1.5
1
0.5
0
Tb.n
Control
MP2+AJ2
MP2+AJ2+sNK
0.5
0.4
0.3
0.2
0.1
0
Tb.Sp
Control
MP2+AJ2
MP2+AJ2+sNK
B
Control
MP2+AJ2
MP2+AJ2+sNK

Figure S5

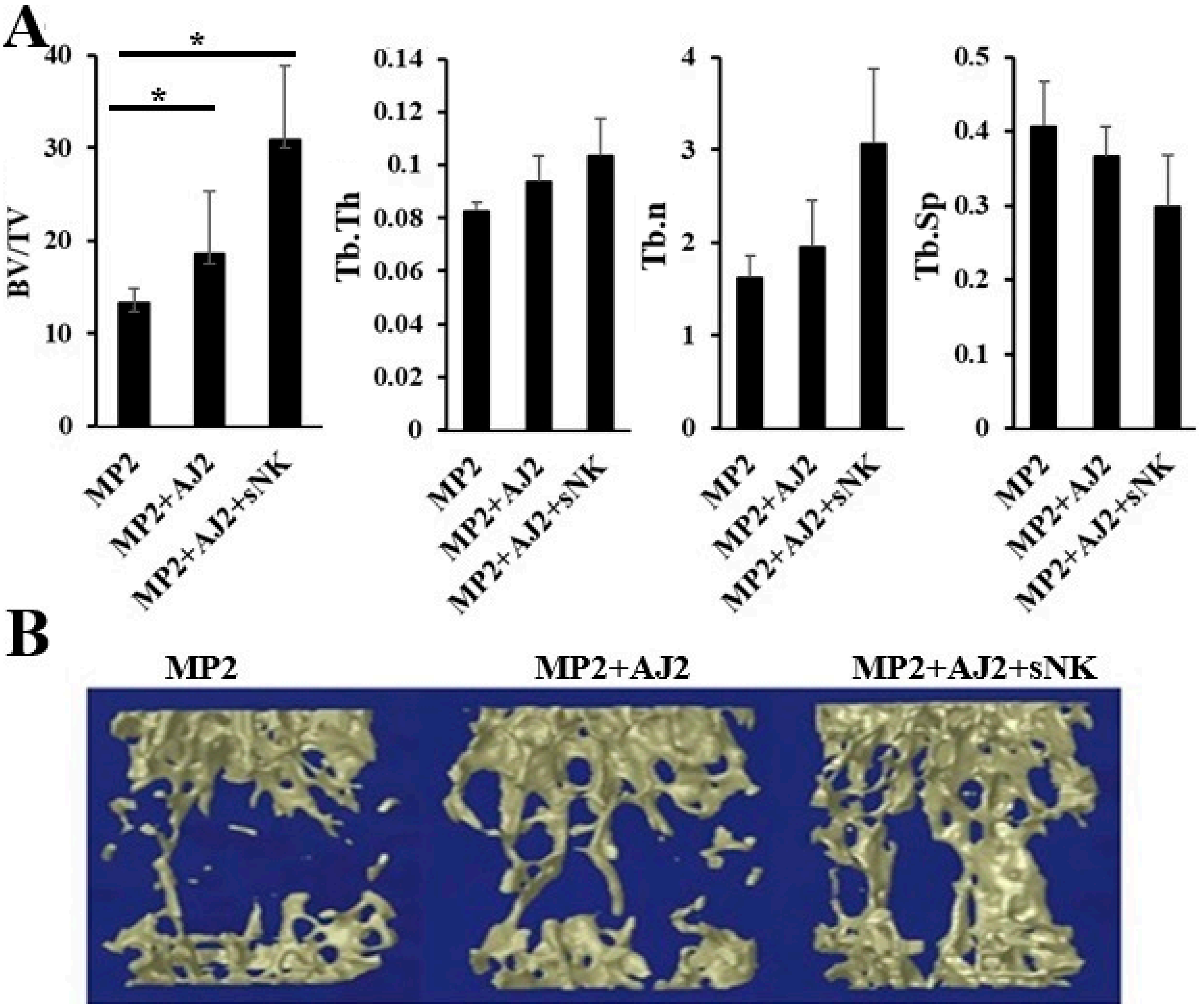
A
BV/TV
Tb.Th
Tb.n
Tb.Sp
MP2
MP2+AJ2
MP2+AJ2+sNK
B
MP2
MP2+AJ2
MP2+AJ2+sNK
